# MRI Radiomic Features: Association with Disease-Free Survival in Patients with Triple-Negative Breast Cancer

**DOI:** 10.1038/s41598-020-60822-9

**Published:** 2020-02-28

**Authors:** Sungwon Kim, Min Jung Kim, Eun-Kyung Kim, Jung Hyun Yoon, Vivian Youngjean Park

**Affiliations:** Department of Radiology and Research Institute of Radiological Science, Severance Hospital, Yonsei University College of Medicine, Seoul, Korea

**Keywords:** Biomarkers, Medical research, Oncology

## Abstract

Radiomic features hold potential to improve prediction of disease-free survival (DFS) in triple-negative breast cancer (TNBC) and may show better performance if developed from TNBC patients. We aimed to develop a radiomics score based on MRI features to estimate DFS in patients with TNBC. A total of 228 TNBC patients who underwent preoperative MRI and surgery between April 2012 and December 2016 were included. Patients were temporally divided into the training (n = 169) and validation (n = 59) set. Radiomic features of the tumor were extracted from T2-weighted and contrast-enhanced T1- weighted MRI. Then a radiomics score was constructed with the least absolute shrinkage and selection operator regression in the training set. Univariate and multivariate Cox proportional hazards models were used to determine what associations the radiomics score and clinicopathologic variables had with DFS. A combined clinicopathologic-radiomic (CCR) model was constructed based on multivariate Cox analysis. The incremental values of the radiomics score were evaluated by using the integrated area under the receiver operating characteristic curve (iAUC) and bootstrapping (n = 1000). The radiomics score, which consisted of 5 selected MRI features, was significantly associated with worse DFS in both the training and validation sets (*p* = 0.002, *p* = 0.033, respectively). In both the training and validation set, the radiomics score showed comparable performance with the clinicopathologic model. The CCR model demonstrated better performance than the clinicopathologic model in the training set (iAUC, 0.844; difference in iAUC, *p* < 0.001) and validation set (iAUC, 0.765, difference in iAUC, *p* < 0.001). In conclusion, MRI-based radiomic features can improve the prediction of DFS when integrated with clinicopathologic data in patients with TNBC.

## Introduction

Breast cancer is a heterogeneous disease, and comprehensive genomic analysis has revealed the existence of four main breast cancer classes^1^, similar to the intrinsic subtypes characterised by microarray-based gene expression profiling^2^. Although each subtype shows different prognosis and response to treatment^3^, such genomic tests are not easily available in much of the world for financial and logistical reasons. Therefore, in clinical practice, surrogate approaches have been developed which incorporate more commonly available immunohistochemical tests for the estrogen receptor (ER) and progesterone receptor (PR) and *in situ* hybridization tests for the human epidermal growth factor receptor 2 (HER2) overexpression or amplification^4^.

Whereas HER2-positive tumors have been the subject of great clinical success due to effective therapeutic targeting of HER2^5^, chemotherapy remains the only established option for triple-negative breast cancers (TNBC). TNBC comprises 10–20% of all breast cancers, and has been associated with early relapse and worse survival^6^. However, there is substantial heterogeneity in the individual outcomes of patients with TNBC^6^. Therefore, many efforts have been made to improve risk stratification in patients with TNBC, including attempts to identify possible imaging biomarkers for this subgroup^7–10^.

During the past five years, radiomics research has shown exponential progress in the field of oncology, and has also been actively applied to breast imaging^11^. Recently, radiomic features observed at preoperative staging magnetic resonance imaging (MRI) were reported to be independent biomarkers for disease-free survival (DFS) in patients with invasive breast cancer^12^. However, the majority of breast cancers included in this previous study were luminal subtype tumors, with the radiomics signature and the triple-negative subtype being identified as independent prognostic factors for DFS^12^. However, the imaging features of TNBC differ from non-TNBC subtypes, and semantic MRI features that are associated with worse survival also differ between breast cancer subtypes^8,13,14^. Therefore, radiomic features that predict survival may differ between TNBC and non-TNBC turmors, and can potentially show better performance if they have been developed from a subgroup made up of only TNBC patients.

Therefore, the purpose of this study was to develop a radiomics score based on MRI features to estimate DFS in patients with TNBC.

## Results

### Patient characteristics and survival outcomes

The median follow-up period was 48.0 months (range, 5.0–80.0 months). Disease recurred in 32 of 228 patients (14.0%) at a median 12.5 months (range, 2.5–60.2 months). Among these 32 patients, 16 (50.0%) had distant recurrence, 12 (37.5) had both distant and locoregional recurrence, and 4 (12.5%) had locoregional recurrence. Thirteen patients died during treatment for recurrence, with a median time to death of 16.5 months (range, 11.0–45.0 months).

The characteristics of the patients in the training and validation sets are shown in Table 1. None of the clinicopathologic variables differed between the two cohorts.

**Table 1:** Patient characteristics in the training and validation set.

Characteristics	Training set (n = 169)	Validation set (n = 59)	*p* value
Age, years*	52.2 ± 12.5	53.7 ± 11.5	0.426
Tumor size on MRI, mm*	27.5 ± 16.0	28.1 ± 16.1	0.789
Pathological T category			0.120
pT1	114 (67.5)	37 (62.7)	
pT2	47 (27.8)	22 (37.3)	
pT3	8 (4.7)	0 (0)	
Pathological N category			0.708
pN0	134 (79.3)	44 (74.6)	
pN1	25 (14.8)	10 (16.9)	
pN2	10 (5.9)	5 (8.5)	
Type of surgery			0.896
Breast-conserving surgery	113 (66.9)	40 (67.8)	
Mastectomy	56 (33.1)	19 (32.2)	
Adjuvant radiation therapy			0.878
No	30 (17.8)	11(18.6)	
Yes	139 (82.2)	48 (81.4)	
Neoadjuvant chemotherapy			0.871
No	125 (74.0)	43 (72.9)	
Yes	44 (26.0)	16 (27.1)	
Adjuvant chemotherapy			0.706
No	59 (34.9)	19 (32.2)	
Yes	110 (65.1)	40 (67.8)	
Histological grade			0.531
1 or 2	61 (36.1)	24 (40.7)	
3	108 (63.9)	35 (59.3)	
Lymphovascular invasion			0.818
No	156 (92.3)	55 (93.2)	
Yes	13 (7.7)	4 (6.8)	

Note.—Unless otherwise noted, data are numbers of patients, with percentages in parentheses.

*Data are means ± standard deviations.

### Feature selection, radiomics score building and validation

Of the extracted radiomic features, we selected 2412 radiomic features with a ICC value greater than 0.75. These features were reduced to five potential predictors with nonzero coefficients in the LASSO Cox regression model based on the 169 patients in the training set. The five predictors consisted of one feature from contrast-enhanced T1-weighted (CE T1W) images and four features from T2-weighted (T2W) images. The radiomics score (Rad-score) was constructed by combining these five features, with a Rad-score calculated for each patient as a linear combination of the selected features that were weighted by their respective coefficients (Supplementary Information).

**Figure 1:**
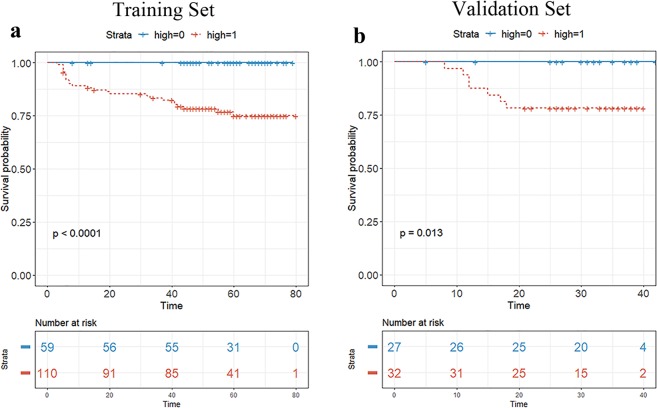
Kaplan-Meier survival analyses were performed according to the radiomics score for patients in the training data set (**a**) and those in the validation data set. (**b**) The validation set was stratified into a low-and high-risk group based on a cut-off value determined in the training data set. A significant association of the radiomics score with DFS was shown in the training data set, which was then confirmed in the validation data set.

Among clinicopathologic factors, tumor size on MRI, pathologic T category, pathologic N category, type of surgery, neoadjuvant chemotherapy, adjuvant chemotherapy, lymphovascular invasion were associated with worse DFS at univariate analysis (Table 2). In multivariate analysis, pathological N category (pN1 vs. pN0, HR 2.906, *p* = 0.035; pN2 vs. pN0, HR 6.622, *p* = 0.001) and lymphovascular invasion (HR 2.566, *p* = 0.046) were identified as independent factors. Multivariate Cox analysis, including these two clinicopathologic independent factors and the Rad-score, confirmed that the Rad-score was an independent factor in both the training set (HR, 78.182, *p* = 0.002) and validation set (HR, 210.516, *p* = 0.033; Table 3). In the Rad-score-only model, the optimal cutoff of the Rad-score for differentiating patients into either the high-risk and low-risk group was obtained in the training data set based on the maximally selected log-rank statistic (log-rank test, *p* < 0.001,). When the validation set was stratified into the high-risk and low-risk groups using the cutoff derived from the training set, the survival curves of the two groups were significantly different (*p* = 0.013) (Fig. 1).

**Table 2:** Survival analysis of DFS according to clinicopathologic variables.

Characteristics	Univariate		Multivariate	
	HR	*p* value	HR	*p* value
Age, years	0.979 (0.950, 1.008)	0.158		
Tumor size on MRI (mm)	1.023 (1.008, 1.039)	0.003	0.993 (0.972, 1.015)	0.532
Pathological T category				
pT1				
pT2	2.749 (1.341, 5.637)	0.006	1.812 (0.775, 4.240)	0.170
pT3	2.901 (0.659, 12.78)	0.159	1.529 (0.316, 7.391)	0.597
Pathological N category				
pN0				
pN1	6.416 (2.826, 14.56)	<0.001	2.906 (1.080, 7.821)	0.035
pN2	16.984 (6.977, 41.34)	<0.001	6.622 (2.099, 20.887)	0.001
Type of surgery				
Breast-conserving surgery				
Mastectomy	3.873 (1.892, 7.926)	<0.001	2.281 (0.963, 5.402)	0.061
Adjuvant radiation therapy				
No				
Yes	1.211 (0.466, 3.146)	0.694		
Neoadjuvant chemotherapy				
No				
Yes	5.231 (2.575, 10.63)	<0.001	1.320 (0.267, 6.528)	0.733
Adjuvant chemotherapy				
No				
Yes	0.220 (0.106, 0.456)	<0.001	0.364 (0.077, 1.719)	0.202
Histological grade				
1 or 2				
3	1.321 (0.626, 2.791)	0.465		
Lymphovascular invasion				
No				
Yes	10.195 (4.881, 21.29)	<0.001	2.566 (1.018, 6.469)	0.046

**Table 3:** Prognostic factors of disease-free survival for the training and validation set in the combined clinicopathologic and radiomic model.

Characteristics	Training set (n = 169)				Validation set (n = 59)			
	Univariate		Multivariate		Univariate		Multivariate	
	HR	*p* value	HR	*p* value	HR	*p* value	HR	*p* value
Rad-score	1.690 (1.305, 2.188)	<0.001	1.546 (1.176, 2.033)	0.002	2.065 (1.226, 3.480)	0.006	1.707 (1.043, 2.795)	0.033
Pathological N category								
pN0								
pN1	6.129 (2.429, 15.460)	<0.001	3.056 (1.078, 8.661)	0.036	7.019 (1.171, 42.080)	0.033	3.659 (0.4741, 28.232)	0.214
pN2	18.821 (6.956, 50.920)	<0.001	7.603 (2.162, 26.735)	0.002	12.468 (1.748, 88.910)	0.012	3.003 (0.3211, 28.078)	0.335
Lymphovascular invasion								
No								
Yes	10.858 (4.754, 24.800)	<0.001	2.950 (1.047, 8.308)	0.041	7.023 (1.351, 36.520)	0.021	3.114 (0.4568, 21.220)	0.246

### Development and validation of the CCR Model

A clinicopathologic model and a CCR model that incorporated the radiomics score and significant clinicopathologic factors in the data set were also established. We evaluated the performance of the three models for prediction of DFS. In the training set, the radiomics score (Rad-score-only model) showed a prognostic performance (iAUC, 0.747 [95% CI: 0.668, 0.824]) comparable with the clinicopathologic model (iAUC, 0.764 [95% CI: 0.669, 0.853]), without statistically significant difference (difference in iAUC: −0.017 [95% CI: −0.127, 0.091]). The highest prognostic performance was observed with the CCR model (iAUC, 0.844 [95% CI: 0.771, 0.912]), which showed a significant improvement in survival prediction compared to the clinicopathologic model (difference in iAUC: 0.080 [95% CI: 0.029, 0.144], *p* < 0.001) (Table 4).

**Table 4:** Comparison of prognostic performance between the clinicopathologic model and combined clinicopathologic and radiomic model.

Set	CP model	Radiomic model	CCR model	Differences between the CCR and CP model (CI)	*p* value
Training set	0.764 (0.669, 0.853)	0.747 (0.668, 0.824)	0.844 (0.771, 0.912)	0.080 (0.029, 0.144)	<0.001
Validation set	0.691 (0.648, 0.719)	0.701 (0.674, 0.725)	0.765 (0.724, 0.790)	0.073 (0.034, 0.114)	<0.001

Note.—Performance values were measured using iAUC, and the values in parentheses are the confidence interval.

*CP model* clinicopathologic model; *CCR model* combined clinicopathologic and radiomic model; *CI* confidence intervals.

When tested in the validation set, the radiomcs score yielded an iAUC of 0.701 (95% CI: 0.674, 0.725). The radiomics score showed comparable performance with the clinicopathologic model (iAUC, 0.691 [95% CI: 0.648, 0.719]), without statistically significant difference (difference in iAUC: 0.009 [95% CI: −0.028, 0.058]). The CCR model (iAUC, 0.765 [95% CI: 0.724, 0.790]) showed significant improvement over the clinicopathologic model for the prediction of DFS in the validation set (difference in iAUC, 0.073 [95% CI: 0.034, 0.114], *p* < 0.001) (Table 4).

The interobserver reproducibility of the extracted radiomic features showed ICC values ranging from 0.329 to 1.00. The finally selected radiomic features showed high ICC values (median, 0.964; range, 0.785, 0.994) (Supplementary Table S1)

## Discussion

In this study we showed that the radiomics score was associated with DFS in both the training and validation data set, and that it remained an independent prognostic factor at multivariate analysis. Furthermore, the CCR model showed a significant improvement over the clinicopathologic model. As the clinicopathologic model was determined by the entire data set (n = 228), this approach would have provided a larger advantage to the clinicopathologic model. Nonetheless, the radiomics score enabled further improvement in DFS prediction, showing that radiomic features do contain additional information that can potentially be used in risk assessment.

In the last few years, researchers have used various approaches when applying MRI-based radiomic features in breast imaging, but most studies have focused on the differential diagnosis of breast lesions, the prediction of pathological characteristics, and response to neoadjuvant chemotherapy^15–22^. Recently, one study reported that a MRI-based radiomics signature was an independent predictor of DFS^12^. However, only 17% of its validation set were TNBC, and the triple-negative subtype remained an independent prognostic factor^12^. TNBC shows different imaging features compared to other breast cancer subtypes, often presenting with areas of intratumoral high T2 signal intensity, lobulated shape, rim enhancement, and smooth margins^14^. Previous studies which investigated the role of radiomics in differentiating breast cancer subtypes have also shown that radiomic features related to lesion shape contribute the most among other features when discriminating TNBC from other subtypes^19,23^. Interestingly, whereas one shape-related radiomic feature was selected in the aforementioned study from Park *et al*.^12^, none of the shape-related features were selected in our study. Our study results may indicate that shape-related features may be less helpful in risk stratification for TNBC.

However, similar to the study of Park *et al*. in which all other radiomic features were T2W-imaging based features, we found that four of the five selected radiomic features in our study were also extracted from T2W images. Another previous study also reported that breast cancers that appear more heterogeneous on T2W images (higher entropy) exhibited poorer recurrence-free survival^24^. In addition, in one study which investigated the performance of a MRI-based radiomics classifier for predicting Ki-67 status in breast cancer, the T2W-imaging-based radiomics classifier significantly predicted Ki-67 status and outperformed the CE T1W-imaging-based rclassifier^16^. As Ki-67 is a well-established prognostic marker in breast cancer, especially in ER-positive breast cancer^25^, this may partially explain the high contribution of T2W images for predicting survival using radiomic features. Our results and previous studies highlight the importance of including T2W images for analysis in radiomics research regarding survival in breast cancer.

In an attempt to improve risk stratification in TNBC, previous studies have aimed to identify MRI features associated with survival, with mixed results^7,8,26^. Whereas rim enhancement was associated with poorer outcome only in TNBC in one study^26^, another reported that rim enhancement was associated with poor distant metastasis-free survival only in hormone receptor-positive or HER2 positive tumors^8^. Compared to semantic imaging features, quantitative radiomic features have the advantage of being less affected by interobserver variability. In our study, we found that the radiomics score was not only significantly associated with DFS in TNBC but also improved its prediction. As the selected independent clinicopathologic factors were based on peritumoral or non-tumor characteristics (pathological N category, lymphovascular invasion), it is likely that tumor-based radiomic features provided complementary information that could further improve the performance of both the clinicopathologic model or the radiomics score alone.

One difficulty with research on TNBC is its relatively small proportion among breast cancer (10–20%)^27^. This is especially true for studies related to survival, which require longer follow-up and thus, are more affected by follow-up loss. In order to construct both the training and validation data set, we included patients without regard to neoadjuvant chemotherapy. Although this approach has been used in previous studies and despite the fact that we included treatment variables in analysis^28,29^, it may be more desirable to construct a radiomics score separately based on neoadjuvant chemotherapy status. Furthermore, as we aimed to compare the performance of the radiomics score with a clinicopathological model for estimating DFS, patients achieving pCR were eventually excluded due to unavailable pathological data. Therefore, our results may not be transferable to patients with TNBC who achieve pCR. Yet, our preliminary study showed that the radiomics score can potentially improve risk stratification in TNBC, and that T2W-imaging-based features provide valuable information regarding survival, similar to other breast cancer subtypes. These results will aid in the design of future studies with larger study populations, more preferably of multi-center design, that will confirm the prognostic role of radiomics for TNBC.

Our study had several limitations. First, this was a retrospective, single-institution study, and selection bias is inevitable. As all images were obtained using the same MRI protocol and scanner, it may be difficult to generalise our results to different settings. In addition, as we did not perform external validation using an independent data set from different institutions, there is a possibility of overfitting. Second, this study had a relatively small sample size and relatively short follow-up period. As TNBC represents a small proportion of primary breast cancers, future multi-center studies with larger study populations and longer follow-up are needed to determine the role of radiomics as a prognostic tool in TNBC patients, including its association with overall survival. Third, lesion segmentation was performed by one radiologist using semiautomatic software. Although we discarded radiomic features with an ICC value of less than 0.75, future software that can perform fully automatic segmentation can help reduce interobserver variability and improve the feasibility of performing radiomics analysis in daily practice. Finally, as MRI is not universally performed for preoperative evaluation of breast cancer, the clinical utility of such MRI-based radiomics features may be potentially limited in actual clinical practice.

In conclusion, our preliminary study shows that the identified radiomics score has the potential to be used as a biomarker for risk stratification in patients with TNBC. The CCR model, which incorporated the radiomics score with clinicopathologic data, showed significant improvement in the prediction of DFS. However, further validation with larger study populations is required to confirm the prognostic role of radiomics in TNBC.

## Methods

### Patients

The institutional review board of Severance hospital approved this retrospective study and the requirement for informed consent was waived. All research was performed in accordance with relevant guidelines/regulations. We identified 342 consecutive women with triple-negative (ie., estrogen-receptor-negative, progesterone receptor-negative, and human epidermal growth factor receptor 2 [HER2]-negative) invasive breast cancer who underwent surgery following preoperative MRI using a 3-T scanner (Discovery MR750w; GE Healthcare) at our institution between April 2012 and December 2016^30,31^.

Of the initial 342 patients, we excluded 114 patients for the following reasons: patients with recurrent breast cancer (n = 23), patients presenting with systemic metastases (n = 5), patients with malignancy other than the primary breast cancer (n = 3), patients who received neoadjuvant chemotherapy prior to MRI (n = 2), patients in whom MRI was performed after vacuum-assisted or excisional biopsy (n = 9), patients with occult breast cancer (n = 1), patients with unavailable pathological variables (n = 61), patients with bilateral breast cancer (n = 5), and patients who were immediately loss to follow-up after surgery (n = 5). Finally, 228 patients (mean age, 53 years; range, 22–85 years) were included in this study. For independent temporal validation, patients who underwent surgery up to April 2015 were assigned to the training set (n =  169, mean age, 52 years [range, 25–85 years]) and subsequent patients were assigned to the validation set (n = 59; mean age, 54 years [range, 22–81 years]).

### Immunohistochemical Staining and Interpretation

Immunohistochemical staining for the ER, PR and HER2 status was performed on tissue slices with standard methods^30,31^. A cut-off value of ≥ 1% positively stained nuclei was used to define ER and PR positivity using the Envision FLEX Kit (DAKO, Glostrup, Denmark). HER2 staining using the Hercep Test TM (DAKO, Glostrup, Denmark) was scored as 0, 1+, 2+, or 3+ according to the American Society of Clinical Oncology (ASCO)/College of American Pathologists (CAP) guidelines^31^. HER2 immunostaining was considered positive when strong (3+) membranous staining was observed whereas cases with 0–1+ were regarded as negative. In cases with a HER2 2+ result, silver *in situ* hybridization (SISH) was performed using the INFORM HER2 Dual ISH DNA Probe Cocktail Assay (Ventana Medical Systems, Tucson, AZ) with an automated slide stainer according to the manufacturer’s protocols. HER2 gene amplification was defined with a HER2 gene/chromosome 17 copy number ratio ≥ 2.0 or a HER2 gene/chromosome 17 copy number ratio < 2.0 with an average HER2 copy number ≥ 6.0 signals/cell according to the ASCO/CAP guidelines.

### Clinicopathologic evaluation and follow-up

Patient age, information on clinical follow-up and on treatment modalities including surgery, radiation therapy, and neoadjuvant or adjuvant chemotherapy were obtained from medical records. The final histopathological results of surgical specimens were reviewed to determine pathological T and N categories, histologic grade and lymphovascular invasion. Tumor size at initial preoperative MRI was obtained from the radiology report. The follow-up protocol for patients is described in the Supplementary Information.

The end point of our study was DFS, which was defined as the time interval from the date of surgery to development of the first evidence of events. Events for determining DFS were events of breast cancer recurrence (locoregional or distant recurrence) or the development of a new primary contralateral breast cancer.

### MRI technique

MRI was performed with a 3 T scanner (Discovery MR750w; GE Healthcare, Milwaukee, WI, USA) with a dedicated phased array breast coil. All patients underwent MRI in the prone position. After obtaining three-plane localizer images, axial T2-weighted (T2W) fast spin-echo images (TR/TE, 4187/102; matrix, 320 × 256 pixels; field of view, 320 × 320 mm; section thickness, (3) and axial T2 STIR images (TR/TE, 5000/70; TI, 200 ms) were obtained. After obtaining axial diffusion-weighted images with a 2D spin-echo echo-planar imaging (EPI) sequence, a T1-weighted (T1W) dynamic contrast-enhanced (CE) sequence was performed. This included one precontrast acquisition and six postcontrast bilateral axial acquisitions (VIBRANT-Flex Dyn. imaging; matrix, 280 × 512 pixels; flip angle, 12 degrees; field of view, 320 × 320 mm; section thickness, 3 mm, no intersection gap) (Supplementary Information).

### MRI preparation for radiomic feature analysis

#### Lesion segmentation and image preprocessing

One breast radiologist (V.Y.P, with 5 years of subspecialty experience in breast imaging) semiautomatically segmented the tumor lesion in early contrast-enhanced T1-weighted images using MIPAV software (Medical Imaging Processing Analysis & Visualization, National Institutes of Health, mipav.cit.nih.gov) and the generated mask was used for CE T1W and T2W images. To evaluate interobserver reproducibility, another breast radiologist (M.J.K, with 16 years of subspecialty experience in breast imaging) independently performed tumor segmentation on 40 randomly chosen lesions. Further details are given in the Supplementary Information.

#### Radiomic feature extraction

Radiomic feature extraction and additional image preprocessing were performed using open source PyRadiomics software (version 2.1.2; Computational Imaging and Bioinformatics Lab, Harvard Medical School)^32^. In this study, a total of 2436 candidate radiomic features were generated from the CE T1W and T2W images including features of shape, histogram, GLCM, GLRLM, GLSZM, GLDM with or without imaging filters (Laplacian of Gaussian, Wavelet). Details are presented in the Supplementary Information.

### Statistical analysis

Patient characteristics were compared between the training and validation set using the Student’s *t*-test for continuous variables and the chi-squared test or Fisher’s exact test for categorical variables.

For feature selection, radiomic features with ICC values of less than 0.75 were removed. We then used the least absolute shrinkage and selection operator (LASSO) method using 10-fold cross validation, to select the most significant features in the training data set^33^. The LASSO is a data analysis method that is suitable for the regression of high-dimensional data. The selected imaging features were then combined into a radiomics score (Rad-score). For each patient, a Rad-score was calculated through a linear combination of selected features weighted by their respective coefficients.

We first assessed the potential association of the radiomics score with DFS in the training set, and then validated it in the validation set. Patients were classified into high-risk or low-risk groups according to the Rad-score, by the maximally selected log-rank statistic^34^ Kaplan-Meier curves were used to analyse DFS between the high-risk and low-risk groups, and log-rank tests were used to compare differences in survival. In addition, to determine clinicopathologic variables associated with DFS, we used the univariate Cox proportional hazards model to analyze the association between clinicopathologic variables in the whole data set (n = 228). Multivariate Cox regression was performed for variables with a *p* value of < 0.1 in the univariate Cox regression analysis.

In addition to the radiomics score (Rad-score-only model), we built a clinicopathologic model and a combined clinicopathologic-radiomics (CCR) model to evaluate the prognostic performance of all three models and demonstrate the value of the radiomics score. The CCR model incorporated the radiomics score and independent clinicopathologic risk factors based on the multivariate Cox analysis. The performance of the CCR model was compared with that of both the Rad-score-only model and clinicopathologic model in the training set and validation set, respectively. Model performance was calculated by using the integrated area under the time-dependent ROC curve (iAUC) based on predicted risks from each model (Supplementary Information).

The interobserver variability of the radiomic features was assessed with the intraclass correlation coefficient (ICC). An ICC value greater than 0.75 was considered to represent good reproducibility^35^. Changes in hazard ratio (HR) were calculated with a 0.1-unit difference in the Rad-score. All statistical and radiomic analyses were performed using the R software (version 3.3.1; R Foundation for Statistical Computing). A two-tailed *p* value of <0.05 was considered statistically significant.

## Supplementary information

Supplementary information.

## Data Availability

The datasets generated during and/or analysed during the current study are available from the corresponding author on reasonable request.
