# Supplementary material for: MRI Radiomic Features: Association with Disease-Free Survival in Patients with Triple-Negative Breast Cancer
Source: Sci Rep. 2020 Feb 28;10:3750. doi: 10.1038/s41598-020-60822-9 (PMC7048756; doi:10.1038/s41598-020-60822-9)
Supplement: Supplementary file 1 — Supplementary information. [file 41598_2020_60822_MOESM1_ESM.pdf]

Title: MRI Radiomic Features: Association with Disease-Free Survival in Patients with Triple-Negative Breast Cancer

Contributing Authors:

Sungwon Kim, MD [1], Min Jung Kim, MD, PhD [1], Eun-Kyung Kim, MD, PhD[1], Jung Hyun Yoon, MD, PhD[1], Vivian Youngjean Park, MD, PhD[1]

Affiliations:

[1]Department of Radiology and Research Institute of Radiological Science, Severance Hospital, Yonsei University College of Medicine, Seoul, Korea

Supplementary Methods 1. Follow-up Protocol

After surgery, patients were followed up with a clinical examination and breast US every 6 months and with mammography, chest radiography, whole-body bone scan, and abdominal US every 12 months after surgery. Breast MR imaging has been increasingly implemented as part of the surveillance protocol in our institution since 2013, and patients underwent surveillance breast MRI instead of US twice , approximately two and five years after surgery. During follow-up, breast MRI and PET-CT were also performed when considered necessary by the referring physician.

Supplementary Methods 2. MRI Technique

MRI was performed with a 3T scanner (Discovery MR750w; GE Healthcare, Milwaukee, WI, USA) with a dedicated phased array breast coil. All patients underwent MRI in the prone position. After obtaining three-plane localizer images, axial T2-weighted (T2W) fast spin-echo images (TR/TE, 4187/102; matrix, 320 x 256 pixels; field of view, 320 x 320

mm; section thickness, 3) and axial T2 STIR images (TR/TE, 5000/70; TI, 200 ms) were obtained. After obtaining axial diffusion-weighted images with a 2D spin-echo echo-planar imaging (EPI) sequence, a T1-weighted (T1W) dynamic contrast-enhanced (CE) sequence was performed. This included one precontrast acquisition and six postcontrast bilateral axial acquisitions (VIBRANT-Flex Dyn. imaging; matrix, 280 x 512 pixels; flip angle, 12 degrees; field of view, 320 x 320mm; section thickness, 3mm, no intersection gap). The length of each dynamic series was 63 seconds. Postcontrast images were obtained immediately after the contrast material was injectedwith no time delay. Therefore, contrast-enhanced images were acquired at approximately 0, 63, 126, 189, 252, and 315 seconds after the start of contrast material administration. Image subtraction was performed after the dynamic series. A gadolinium-based contrast agent (Dotarem; Guerbet, Paris, France/ Magnevist; Berlex Laboratories, Wayne, NJ, USA/ Gadovist; Bayer Schering Pharma, Berlin, Germany) was injected into an antecubital vein at a dose of 0.2 cc/kg of body weight and at a rate of 2mL/s, using an automated injector and followed by a 20-mLsaline flush.

Supplementary Methods 3. Lesion Segmentation and Image Preprocessing

CE T1W and T2W STIR images were downloaded from the Picture Achiving Communication System in a Digital Imaging and Communications in Medicine format. Early CE T1-weighted images obtained approximately 63 seconds after administration of contrast material began were assessed. One breast radiologist (V.Y.P, with 5 years of subspecialty experience in breast imaging) semiautomatically segmented the tumor lesion in contrast-enhanced T1-weighted images using MIPAV software (Medical Imaging Processing Analysis & Visualization, National Institutes of Health, mipav.cit.nih.gov) and the generated mask was used for CE T1W and T2W images. In the case of multifocal or multicentric disease, the ROI

was drawn around the largest tumor. To evaluate interobserver reproducibility, another breast radiologist (M.J.K, with 16 years of subspecialty experience in breast imaging) independently performed tumor segmentation on 40 randomly chosen lesions. All volumes (CE T1W and T2W) were corrected for B0 inhomogeneity with N4 bias field correction, as implemented in Slicer 4 (https://www.slicer.org). Voxels in each MR image volume were resampled to an isotropic voxel size of 1.0 × 1.0 × 1.0 mm^3 to minimize the effects of different MRI conditions. SI was normalized using the histogram matching method of simple ITK (Insight Segmentation and Registration Toolkit, http://simpleitk.org) with grayscale discretisation level of 64.

Supplementary Methods 4. Extraction Methodology for Radiomic Features

Radiomic features were extracted using open source PyRadiomics software (version 2.1.2; Computational Imaging and Bioinformatics Lab, Harvard Medical School). Extracted feature classes consisted of the first-order features, shape features, the second-order features, and two high-order filters — the Laplacian of Gaussian (LoG) and Wavelet filter. First-order statistics described the distribution of voxel intensities within the image region defined by the mask with commonly used and basic metrics. The second-order features consisted of Gray Level Co-occurrence Matrix (GLCM) Features, Gray Level Size Zone Matrix (GLSZM) Features, Gray Level Run Length Matrix (GLRLM) Features, Neighboring Gray Tone Difference Matrix (NGTDM) Features, and Gray Level Dependence Matrix (GLDM) Features. Applied filter classes were Laplacian of Gaussian (LoG) and Wavelet filter. The LoG filter is an edge enhancement filter, which emphasizes areas of gray level change, with a sigma defining how coarse the emphasized texture should be. A low sigma emphasizes on fine textures (change over a short distance), where a high sigma value emphasizes coarse textures (gray level change over a large distance). Five sigma values were used in this study (1 mm, 2 mm, 3 mm, 4 mm,

and 5 mm). Wavelet filtering yields 8 decompositions per level (all possible combinations of applying either a high or a low pass filter in each of the three dimensions). The following were extracted from the lesion: 14 shape features, 18 first-order features, 68 second-order features with LoG filters with 5 sigmas, and 8 combinations of the wavelet filter. This led to 1218 features for each sequence and 2436 features for all sequences of T1 CE and T2.

Reference)

Kim, S. et al. Radiomics on Gadoxetic Acid-Enhanced Magnetic Resonance Imaging for Prediction of Postoperative Early and Late Recurrence of Single Hepatocellular Carcinoma. Clin Cancer Res 25, 3847-3855, https://doi.org/10.1158/1078-0432.Ccr-18-2861 (2019).

Supplementary Methods 5. Selection of Radiomics Features

Radiomic features were selected using the least absolute shrinkage and selection operator (LASSO) method to select the most significant features in the training data set. The statistical analyses were performed using R software version 3.5.3 (http://www.R-project.org), where the package ‘glmnet’ was used to apply the LASSO method. Among the selected radiomic features, 24 features with a ICC value less than 0.75 were removed. Finally, one feature from CE T1W images and four features from T2W images were selected and then combined into a radiomics score. A radiomic score (Rad-score) was computed for each patient as a linear combination of selected features that were weighted by their respective coefficients.

Supplementary Methods 6. Statistical Analysis: iAUC

The iAUC is the weighted mean of the AUC over a follow-up period and is used to measure a model’s performance in survival prediction. The iAUC values were calculated by using the “riskset ROC” R package. iAUC differences were calculated by using the

bootstrapping method, in which resampling was performed 1000 times. The difference was considered to be significant if the 95% CI did not include 0.

Supplementary Methods 7. Selected Radiomic Features

The five radiomic features with nonzero coefficients in the LASSO Cox regression model are as follows. The suffix *.1* means that the feature was extracted from contrast-enhanced T1-weighted images, and the suffix *.2* means that the feature was extracted from T2-weighted STIR images.

[1] "wavelet.LHH_gldm_SmallDependenceLowGrayLevelEmphasis.1"

[2] "log.sigma.5.0.mm.3D_glszm_SizeZoneNonUniformityNormalized.2"

[3] "wavelet.HLH_glszm_GrayLevelNonUniformityNormalized.2"

[4] "wavelet.HHL_gldm_DependenceNonUniformityNormalized.2"

[5] "wavelet.LLL_glcm_Imc2.2"

Calculation Formula for the Radiomic Score (Rad-score)

y = -4.03031137 * (wavelet.LHH_gldm_SmallDependenceLowGrayLevelEmphasis.1) +
-0.94195174 * (log.sigma.5.0.mm.3D_glszm_SizeZoneNonUniformityNormalized.2) +
0.06084888 * (wavelet.HLH_glszm_GrayLevelNonUniformityNormalized.2) +
-6.20484995 * (wavelet.HHL_gldm_DependenceNonUniformityNormalized.2) +
-2.17930236 * (wavelet.LLL_glcm_Imc2.2)

Supplementary Methods 8.

Results from comparing the performance of the clinicopathologic model and the combined clinicopathologic-radiomics (CCR) model using the likelihood ratio test are also

presented in Table S2. The likelihood ratio test is a widely used method of determining model suitability when comparing the two models. There were statistically significant differences between the CCR and CP models in both the training and validation sets. Statistical analysis was performed using the R software (version 3.6.1; R Project for Statistical Computing).

Table S1 Radiomic Features with Nonzero Coefficients at LASSO Cox Regression and Interobserver Reproducibility

**Features**	**ICC value**
wavelet.LHH_gldm_SmallDependenceLowGrayLevelEmphasis.1	0.951
log.sigma.5.0.mm.3D_glszm_SizeZoneNonUniformityNormalized.2	0.785
wavelet.HLH_glszm_GrayLevelNonUniformityNormalized.2	0.964
wavelet.HHL_gldm_DependenceNonUniformityNormalized.2	0.994
wavelet.LLL_glcm_Imc2.2	0.997

Table S2 Likelihood ratio test for comparison of goodness of fit between the clinicopathologic, combined clinicopathologic and radiomic model

**Set**	**CP model**	**Radiomic model**	**CCR model**	**Differences between the CCR and CP model**	***p* value**
Training set	35.21 df=3	19.74 df=1	47.66 df=4	12.45 df=1	<.0001
Validation set	8.31 df=3	10.67 df=1	15.16 df=4	6.85 df=1	0.009

Note.— Likelihood ratio test statistics and degree of freedom were calculated using Cox proportional hazard regression.

CP model clinicopathologic model; *CCR model* combined clinicopathologic and radiomic model; *df* degree of freedom
